# Enteroscopic Management of Ectopic Varices in a Patient with Liver Cirrhosis and Portal Hypertension

**DOI:** 10.1155/2016/2018642

**Published:** 2016-08-10

**Authors:** G. A. Watson, A. Abu-Shanab, R. L. O'Donohoe, M. Iqbal

**Affiliations:** St. Vincent's University Hospital, Dublin 4, Ireland

## Abstract

Portal hypertension and liver cirrhosis may predispose patients to varices, which have a propensity to bleed and cause significant morbidity and mortality. These varices are most commonly located in the gastroesophageal area; however, rarely ectopic varices may develop in unusual locations outside of this region. Haemorrhage from these sites can be massive and difficult to control; thus early detection and management may be lifesaving. We present a case of occult gastrointestinal bleeding in a patient with underlying alcoholic liver disease where an ectopic varix was ultimately detected with push enteroscopy.

## 1. Introduction

Variceal bleeding is a common complication seen in patients diagnosed with liver cirrhosis and portal hypertension, and it is associated with high mortality rates. The origin of bleeding can often be detected by direct visualization using endoscopic procedures or radiologically. However, in rare instances the source of bleeding may not be immediately apparent, and it is important to consider the possibility of ectopic varices, located in an area outside of the more common gastroesophageal region. Clinical awareness and suspicion are paramount as bleeding from these sites may be life-threatening and immediate intervention is critical. We present a case of gastrointestinal bleeding in a patient with underlying alcoholic liver disease where the source of bleeding was initially unclear; however, push enteroscopy detected an ectopic varix at the site of a previous hepaticojejunostomy.

## 2. Case Report

A 44-year-old gentleman was transferred to our specialist liver unit from a peripheral hospital with ongoing melaena on a background of alcoholic cirrhosis with portal hypertension. He initially presented with generalised abdominal pain and nausea. His haemoglobin on admission to the peripheral hospital was found to be 7.5 g/dL. His background was significant for chronic pancreatitis and subsequent type 2 diabetes mellitus. Furthermore, three years ago he developed a biliary stricture after laparoscopic cholecystectomy that required multiple biliary stents and ultimately a hepaticojejunostomy.

Physical examination was unremarkable except for mild pallor, palmar erythema, and moderate ascites. Routine blood tests on transfer to our unit are shown in Table 1.

He received multiple blood transfusions throughout his admission (eight in the peripheral hospital, requiring a further twenty-six units in our unit). He was also treated with octreotide and terlipressin due to a high suspicion of portal hypertension induced gastrointestinal haemorrhage.

**Figure 1:**
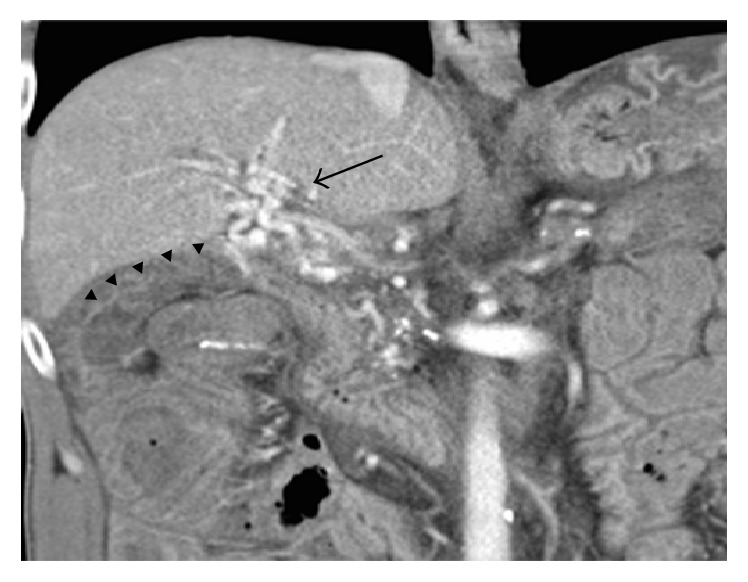
Coronal reformat of the portal venous phase of a multiphase CT liver examination shows an occluded main portal vein with cavernous transformation (arrow). The efferent limb of the hepaticojejunostomy (arrowheads) can be seen extending away from the lateral aspect of the venous collaterals.

An oesophagogastroduodenoscopy (OGD) was performed on admission to the peripheral hospital and showed mild oesophagitis and grade 1 varices. CT angiogram the following day reported no active bleeding. A repeat OGD after transfer to our unit confirmed grade 1 oesophageal varices and portal hypertensive gastropathy, with no source of bleeding identified. A CT four-phase liver examination was performed and showed a nodular liver and occlusion of the main portal vein with cavernous transformation (Figure 1).

During this period the patient continued to have intermittent melaena, up to four times daily, passing circa 500 mL of fresh blood. Haemoglobin levels continued to fall and dropped to 5.4 g/dL on day 10 of admission; however, the patient remained haemodynamically stable. A radiolabeled red blood cell nuclear imaging scan was performed during this period of active haemorrhage; however, the origin of bleeding remained elusive.

The following day a push enteroscopy was attempted using a pediatric colonoscope and was successfully passed to the jejunal loop as far as the hepaticojejunostomy. An area of mucosal erythema with prominent vessels as well as a fresh clot was seen adjacent to the hepaticojejunostomy, features consistent with an ectopic varix.

The case was discussed at length at our multidisciplinary team (MDT) meeting. As the option of TIPS (transjugular intrahepatic portosystemic shunt) was ruled out due to portal vein thrombosis, the feasibility of embolization for presumably ectopic variceal bleed was discussed with our interventional radiologists; however, as there was no obvious source of bleeding identified on previous scans it was difficult to decide on the optimal course of management.

**Figure 2:**
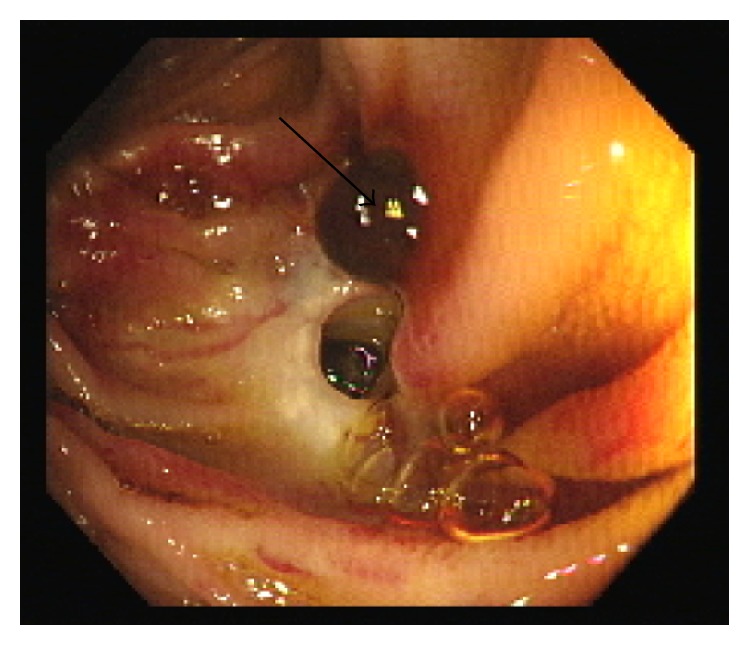
Nipple sign of the ectopic varix at the site of the hepaticojejunostomy (arrow).

**Figure 3:**
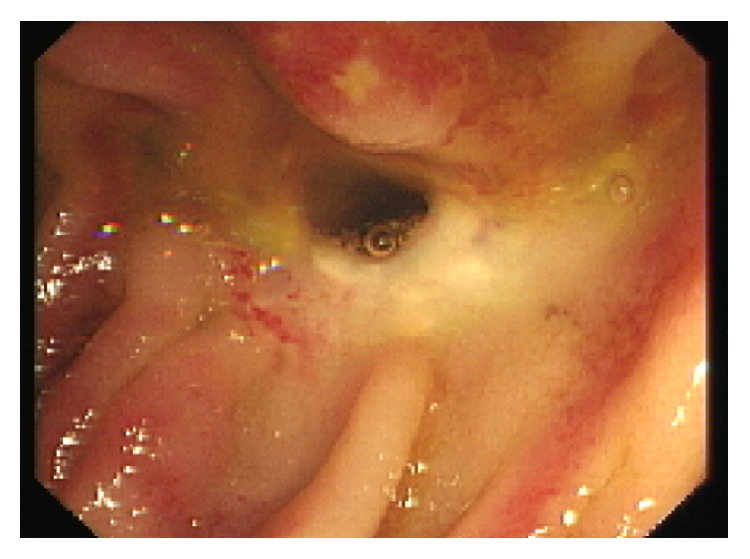
Healed ectopic varix at the site of the hepaticojejunostomy with signs of injection.

Ultimately the overall consensus was that push enteroscopy could be attempted again with the intention of injecting Histoacryl into this ectopic varix at the site of the hepaticojejunostomy. Four days later the procedure was performed and the ectopic varix which was the source of bleeding was finally visualized and identified in the form of a nipple sign at the site of hepaticojejunostomy (Figure 2). Due to its difficult position, the site was injected with Histoacryl and lipiodol. An X-ray of the abdomen was requested and displayed a radiopaque density in the right upper quadrant consistent with filling of the contour of the ectopic varix adjacent to the surgical clips and represented the site of injection (Figure 3).

**Figure 4:**
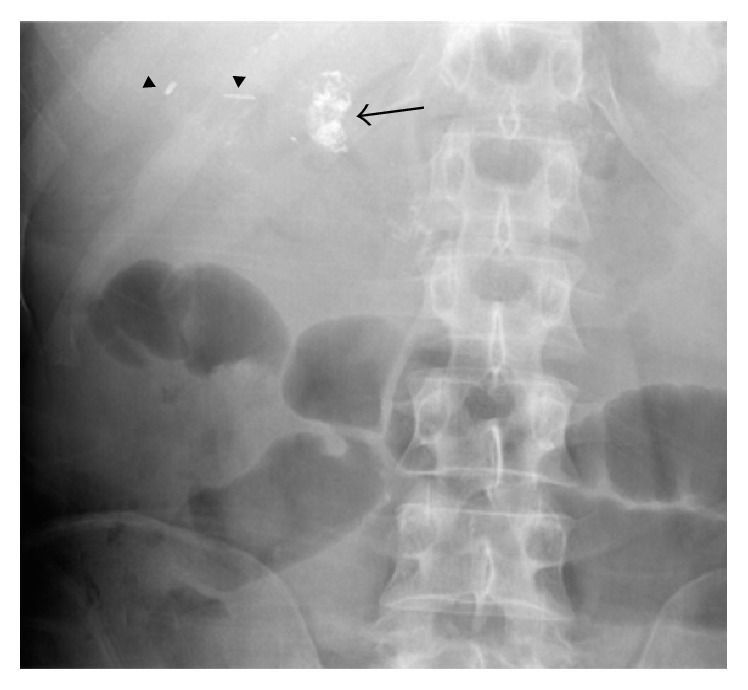
Plain radiograph of the abdomen following injection of the ectopic varix shows radiopaque embolic material (arrow) medial to surgical clips (arrowheads) positioned inferiorly to the right lobe of the liver.

A repeat enteroscopy was performed the following week and clearly displayed a healed ectopic varix at the site of hepaticojejunostomy with signs of injection (Figure 4). Prior to discharge, management options in the case of future rebleeding were discussed at our multidisciplinary meeting (MDM). The general consensus was that while surgical intervention would be an option in the future, it would be associated with a significant risk of morbidity and mortality. It was thus decided that in the case of recurrent bleeding we would again consider endoscopic management.

## 3. Discussion

Portal hypertension and liver cirrhosis may predispose patients to varices, most commonly located in the gastroesophageal area. Ectopic varices may be described as abnormally dilated submucosal vessels located in unusual locations outside of this area. These rare portosystemic collaterals are responsible for 5% of all variceal bleeds [1]. Haemorrhage can be massive with mortality reaching up to 40% [2]; thus clinical awareness and suspicion are crucial in early detection and management in cases where the source of bleeding remains uncertain.

Norton and colleagues reviewed 169 cases of bleeding ectopic varices from different origins. In 26% of cases the source of bleeding was a peristomal varix. Another 17% were duodenal, 17% were jejunal and ileal, 14% were colonic, 9% were peritoneal, 8% were rectal, and a small minority of bleeding varices originated from rare sites such as the ovary and vagina [3]. In another study of 37 patients with liver cirrhosis who underwent capsule endoscopy, 8.1% of patients were found to have small bowel varices [4]. Anorectal varices have been reported in approximately 44% of patients with cirrhosis; however, only a small number become symptomatic [5].

Risk factors for developing varices in locations outside of the more common zones include prior abdominal surgery, and varices have been known to develop in unusual locations such as the urinary bladder, ovaries, and bare area of the liver due to adhesions and in cases of extrahepatic portal hypertension [1, 2, 6, 7]. Ectopic varices may also develop in the absence of portal hypertension due to congenital anomalous portosystemic anastomoses, abnormal vessel structures, arteriovenous fistulae, and rare familial conditions or in relation to thrombosis [8–12].

Patients usually present with hematemesis, melaena, or lower gastrointestinal bleeding, which may range from mild spotting to gross, life-threatening haemorrhage [13]. While initial medical management aims to stabilise the patient haemodyamically with blood transfusions and splanchnic vasoconstrictors such as terlipressin, emergent upper gastrointestinal endoscopy has been the cornerstone of first-line management in patients presenting with gross upper gastrointestinal bleeding. Endoscopic band ligation (EBL) and endoscopic injection sclerotherapy (EIS) have been successfully used in controlling haemorrhage from duodenal [14, 15], jejunal [16], colonic [17], anorectal [18], and stomal varices [19].

To date there are no set guidelines for managing bleeding ectopic varices. Various factors may influence decision-making such as the location of haemorrhage, clinical presentation, and the underlying medical disorder. Akhter and Haskal [2] provided a comprehensive review of the current therapeutic modalities available and recent advances in managing ectopic variceal bleeding, including double balloon enteroscopy and transcatheter embolization or sclerotherapy, with or without portosystemic decompression, that is, transjugular intrahepatic portosystemic shunts (TIPS) [2].

In addition, other modalities outside of the acute setting have proven to be valuable adjuncts. Capsule endoscopy has been successful in visualizing jejunal and small bowel varices [2, 4, 20] while push enteroscopy can also navigate the small bowel and allow for intervention, as was the case in our patient [1, 2, 16, 21]. CT, CT angiography, and CT enteroclysis have all been used for successful diagnosis of duodenal [22, 23] and colonic varices [24]. While technetium TC-99m red blood cell scintigraphy was explored in this case and in previous settings [25], its definitive role in management remains uncertain.

The role of TIPS in the management of bleeding ectopic varices in cirrhotics caused by intrahepatic portal hypertension has frequently been publicised [20, 26]. TIPS have been shown to be more effective in preventing rebleeding from oesophageal varices than endoscopic methods [27, 28]. This means of intervention is often reserved for patients without a history of decompensation (e.g., high MELD (Model of End Stage Liver Disease) score, encephalopathy, and ischaemic liver disease).

Finally, surgical intervention remains an option that is employed less often due to the high risk of morbidity and mortality in patients with liver disease but may be considered a salvage option in a select cohort of patients where previous management strategies have failed.

## 4. Conclusion

Variceal haemorrhage is a feared complication in cirrhotic patients. Its origin is often restricted to the gastroesophageal region; however, development of ectopic varices may occur and clinical suspicion is critical when presented with a patient with obscure haemorrhage with a prior history of hepaticojejunostomy.

Management of ectopic variceal bleed remains uncertain and has only been described in small case reviews and reports to date. We recommend a multidisciplinary approach, which is crucial in guiding decisions regarding management, both in the short term and in the long term. Interventional radiology and surgery should be considered in the event of massive haemorrhage; however, alternative techniques, such as push enteroscopy, can have a big role as an adjunct outside of the acute setting.

## Figures and Tables

**Table 1:** Blood panel. WCC (white cell count), Hb (haemoglobin), MCV (mean corpuscular volume), Hct (haematocrit), Plts (platelets), INR (international normalised ratio), Na+ (sodium), K+ (potassium), Cl− (chloride), Ca2+ (calcium), Inorg PO^4^ (inorganic phosphate), Mg2+ (magnesium), Alb (albumin), BR (bilirubin), Alk Phos (alkaline phosphatase), GGT (gamma glutamyl transpeptidase), ALT (alanine transaminase), AST (aspartate aminotransferase), and CRP (C-reactive protein).

Blood panel			
WCC 15.6 × 10^∧^9/L	Na+ 124 mmol/L	Alb 19 g/L	Ca2+ 1.8 mmol/L
Hb 8.1 g/dL	K+ 4.2 mmol/L	BR 7 *µ*mol/L	Inorg PO^4^ 0.95 mmol/L
MCV 83 fL	Cl− 92 mmol/L	Alk Phos 5 iu/L	Mg2+ 0.66 mmol/L
Hct 0.227 L/L	Urea 5.8 mmol/L	GGT 8 iu/L	
Plts 134 × 10^∧^9/L	Creatinine 54 *µ*mol/L	ALT 15 iu/L	CRP 23.4 mg/L
INR 0.91		AST 16 U/L	
